# Visual Quantitative Detection of Delamination Defects in GFRP via Microwave

**DOI:** 10.3390/s23146386

**Published:** 2023-07-13

**Authors:** Xihan Yang, Yang Fang, Ruonan Wang, Yong Li, Zhenmao Chen

**Affiliations:** State Key Laboratory for Strength and Vibration of Mechanical Structures, Shaanxi Engineering Research Centre of NDT and Structural Integrity Evaluation, School of Aerospace Engineering, Xi’an Jiaotong University, Xi’an 710049, China; yxihan@stu.xjtu.edu.cn (X.Y.); w2502209459@stu.xjtu.edu.cn (R.W.); yong.li@mail.xjtu.edu.cn (Y.L.)

**Keywords:** nondestructive testing, microwave imaging, quantitative characterization, GFRP

## Abstract

Glass Fiber reinforced polymers (GFRPs) are widely used and play an important role in modern society. The multilayer structure of GFRPs can lead to delamination defects during production and service, which can have a significant impact on the integrity and safety of the equipment. Therefore, it is important to monitor these delamination defects during equipment service in order to evaluate their effects on equipment performance and lifespan. Microwave imaging testing, with its high sensitivity and noncontact nature, shows promise as a potential method for detecting delamination defects in GFRPs. However, there is currently limited research on the quantitative characterization of defect images in this field. In order to achieve visual quantitative nondestructive testing (NDT), we propose a 2D-imaging visualization and quantitative characterization method for delamination defects in GFRP, and realize the combination of visual detection and quantitative detection. We built a microwave testing experimental system to verify the effectiveness of the proposed method. The results of the experiment indicate the effectiveness and innovation of the method, which can effectively detect all delamination defects of 0.5 mm thickness inside GFRP with high accuracy, the signal-to-background ratio (SBR) of 2D imaging can reach 4.41 dB, the quantitative error of position is within 0.5 mm, and the relative error of area is within 11%.

## 1. Introduction

Glass fiber reinforced polymer (GFRP) is a lightweight, high specific strength, corrosion-resistant, electrothermally insulating, and transient ultra-high temperature resistant composite material that is widely used in critical structures across various engineering fields, such as aerospace, energy and power, and transportation [1,2,3]. However, due to its laminated structure, GFRPs are susceptible to interlaminar shear stress during processing, assembly, and service, which can result in internal delamination defects that compromise the structural integrity and safety of the material [4,5]. These defects can include delamination between layers and separation of panels, which can lead to a range of potential consequences, such as reduced load-bearing capacity, increased risk of catastrophic failure, and compromised service life. Therefore, it is essential to conduct nondestructive testing and quantitative characterization of GFRP internal delamination defects in order to ensure engineering safety. 

Nondestructive testing (NDT) methods for GFRPs currently include ultrasonic testing [6,7], infrared imaging testing [8], radiographic testing [9], and terahertz testing [10,11,12]. While each method has its own advantages in actual detection, there are also limitations to consider. Ultrasonic detection cannot achieve noncontact detection, and the choice of coupling agent can greatly impact the detection signal. Radiographic detection equipment is expensive and may not provide enough penetration depth for thicker test pieces. Infrared imaging methods are susceptible to signal interference from surrounding environment radiation. Terahertz detection is limited by its penetration depth, making it challenging to detect thicker workpieces. 

Microwave imaging nondestructive testing is a technique that utilizes the difference in microwave response of defects and dielectric materials. It achieves imaging reconstruction and defect characterization through signal processing and parameter extraction from the reflected signal of the target. It is widely used in nonmetal detection and multilayer structure detection due to its high penetration capability, high resolution, large detection depth, noncontact nature, and high efficiency [13,14]. Currently, there are two primary methods for detecting internal defects in nonmetallic materials. The first method is microwave imaging detection, which utilizes imaging algorithms such as wave number domain imaging [15,16], holographic imaging [17], time domain reflectometry [18], and other methods such as unsupervised machine learning methods [19,20]. Microwave imaging detection methods enable visual detection of defects. The second method is microwave coefficient detection, which involves processing the amplitude and phase of the echo signal [21,22,23,24]. By analyzing these characteristics, the location, size, and other parameters of defects can be derived. Microwave coefficient detection methods allow for quantitative assessment of defects. While current microwave nondestructive testing research has achieved significant results in detection and imaging of microscopic defects, there are still gaps in the following areas of research. Firstly, in signal processing algorithms, the current imaging preprocessing filtering and clutter suppression processing is insufficient, leading to poor contrast and indistinguishable defect characteristics. Secondly, in imaging algorithms, the traditional method of feature extraction primarily focuses on imaging, resulting in suboptimal utilization of electromagnetic wave information and low imaging quality. Moreover, the current studies predominantly focus solely on detecting internal defects in GFRP and do not have automated quantitative characterization of defects. To address this issue and effectively detect and accurately characterize internal delamination defects in GFRP, we propose a method based on spectral reconstruction that allows for fast imaging and precise quantitative characterization.

Firstly, in order to suppress strong interference signals and enhance target features, we propose a clutter suppression method based on adaptive singular value decomposition. This method addresses the problem of inaccurate signal subspace division by traditional SVD methods and improves the signal-to-noise ratio (SBR) by adjusting the singular value sequence according to the image standard deviation. Images with good contrast and high quality serve as the foundation for achieving accurate quantitative assessment of defects. Secondly, to obtain a 2D microwave image of the target, we developed a wave spectrum reconstruction algorithm that enables visual detection of the target. The algorithm is based on the 2D Fourier transform and reconstructs the signal distribution at the target plane by performing phase compensation on the signal in the wave number domain. Thirdly, in order to quantitatively characterize defects, we developed the OTSU image segmentation algorithm to accurately separate the target from the background. This algorithm utilizes grayscale traversal to search for the optimal threshold value that maximizes the interclass variance between the target and background regions. By applying the Regionprops function, the pixel value distribution of the defect area is calculated, allowing us to obtain the location and area information of the defect and achieve precise characterization of the internal layered defects of GFRP. Finally, we built a microwave testing experimental system and scanned the designed specimens to verify the effectiveness of the proposed method and analyze its performance. 

## 2. Methods of Spectral Reconstruction Imaging and Quantitative Characterization

**Figure 1:**
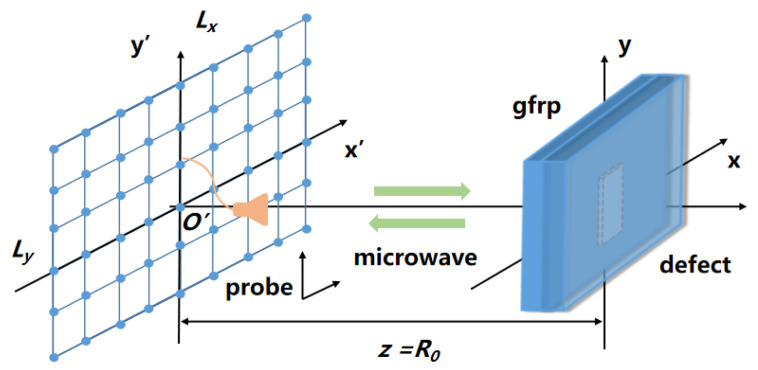
Schematic diagram of 2D scanning microwave detection.

As illustrated in Figure 1, there is continuous movement of the control probe along the x′ and $y^{\prime }$ directions, transmitting and receiving microwave signals within the scanning range $L_{x}\times L_{y}$. The GFRP specimen contains delamination defects and is located at a distance $R_{0}$ from the scanning plane where the control probe is positioned. During the 2D scanning process, the scattering at each sampling position is recorded as a matrix, with each element of the matrix representing the complex echo signal $s(x^{\prime },y^{\prime })$ of a specific sampling coordinate point.

In the actual SISO (single-input single-output) testing, the echo signal $s(x^{\prime },y^{\prime })$, i.e., S11 parameter collected by a vector network analyzer (VNA), represented by Equation (1), is related to various parameters such as the signal frequency f, the specimen thickness d, and the reflection coefficient Γ at the heterogeneous interface. The value of Γ can be obtained from the wave impedance $Z_{s}$, where $Z_{s}$ is a function of magnetic permeability μ and dielectric constant ε. As ε of air and GFRP materials are distinct, when the microwave signal propagates to the interface between air and GFRP, it will be reflected [25]. Therefore, when there are delamination defects inside GFRP, the echo signal $s(x^{\prime },y^{\prime })$ will be changed.

(1) $$S11=\frac{\Gamma (1-e^{2jkd})}{1-\Gamma^{2}e^{2jkd}}\;\Gamma =\frac{Z_{S}-1}{Z_{S}+1}\;Z_{S}=\sqrt{\frac{\mu }{\varepsilon }}$$

**Figure 2:**
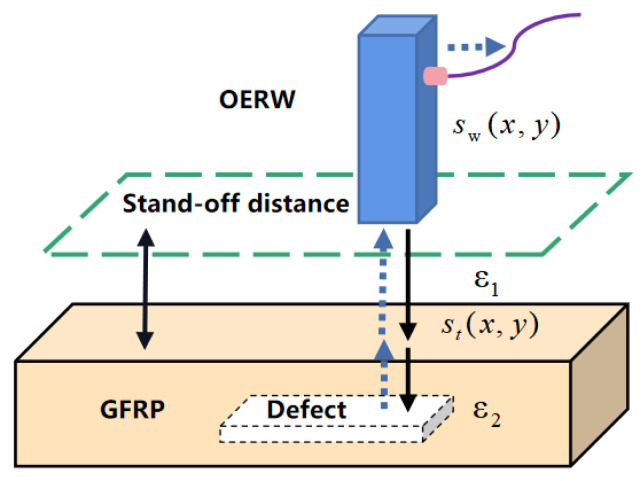
Principle of microwave signal reflection.

As illustrated in Figure 2, when the probe irradiates the GFRP specimen, the signal propagates through the air layer and reaches the surface of the GFRP specimen, and a portion of the signal is reflected back, while another portion continues to propagate through the air–GFRP interface and into the GFRP specimen. As the signal encounters internal delamination defects, it is reflected once again, and the received signal is a combination of coupled and reflected signals, which is then picked up by the probe and recorded by the system as $s(x^{\prime },y^{\prime })$.

$s(x^{\prime },y^{\prime })$ consists of two main components:

(2) $$s(x^{\prime },y^{\prime })=s_{w}(x^{\prime },y^{\prime })+s_{t}(x^{\prime },y^{\prime })$$

The signal $s_{w}(x^{\prime },y^{\prime })$ represents the interference signal, which includes the echo signal at the probe antenna coupling, as well as any other unwanted signals such as direct wave. The probe-coupled reflected wave is the signal caused by the impedance mismatch at the connection when the microwave propagates from VNA to the probe through a cable. Direct wave refers to the microwave signal reflected on the surface of the GFRP specimen when the microwave is emitted from the probe through the air. $s_{t}(x^{\prime },y^{\prime })$ represents the target signal. In order to achieve 2D imaging of the internal delamination defects of GFRP, the target signal is therefore analyzed. As depicted in Figure 1, the coordinates of the probe are represented as $\left(x^{\prime },y^{\prime }\right)$, while the target image function of the measured target is denoted by g(x,y). Then the echo signal at $\left(x^{\prime },y^{\prime }\right)$ is

(3) $$s_{t}(x^{\prime },y^{\prime })=g(x,y)\cdot \exp(-2j\pi \cdot 2Rf/c)$$

(4) $$R=\sqrt{{\left(x^{\prime }-x\right)}^{2}+{\left(y^{\prime }-y\right)}^{2}+R_{0}{}^{2}}$$

The primary objective of 2D imaging is to effectively reconstruct the 2D scattering distribution and determine the target image function g(x,y) [26]. To achieve quantitative characterization of defects, the function g(x,y) is first normalized to function $g^{\prime }(x,y)$. Then, the target region A of the 2D image $g^{\prime }(x,y)$ is separated using threshold segmentation. Finally, the image function $g^{\prime }(x,y)[(x,y)\in A]$ is binarized to function g(x,y)[(x,y)∈A] to enable visual detection of defects. To achieve visual detection and quantitative characterization of internal delamination defects in GFRP, a three-part algorithm based on spectral reconstruction was developed. The algorithm consists of clutter suppression, 2D imaging, and quantitative characterization.

### 2.1. Adaptive Singular Value Decomposition-Based Clutter Suppression

In the processing of the echo signal, achieving high-quality 2D imaging of the target requires initial clutter suppression to improve the signal-to-noise ratio (SNR). Common clutter suppression methods include coherent processing filtering, time-domain filtering, and wavelet denoising. The approach of clutter suppression based on singular value decomposition (SVD) is a subspace algorithm that involves decomposing the signal vector space into two subspaces, where one is predominantly dominated by the target signal and the other by the noise signal. Clutter suppression is achieved by eliminating the signal vector components belonging to the noise-dominated subspace [27]. Let the echo signal $s(x^{\prime },y^{\prime })$ at each sampling point form an m×n dimensional matrix S of rank r. SVD of S is expressed as follows:

(5) $$S=U\left[\begin{array}{cc} \sum & 0\\ 0 & 0 \end{array}\right]V^{H}$$

where U and V are m×m, n×n orthogonal matrices, respectively (*m*, *n* sampling points). ∑ is an r×r dimensional diagonal array whose diagonal elements are the nonzero singular values $\sigma_{i}$ of matrix S and are arranged in nonincreasing order, i.e., $\sigma_{1}\geq \sigma_{2}\geq \cdots \geq \sigma_{r}$. Since the rank of matrix S is r, the streamlined form of the singular value decomposition of S can be obtained by removing the zero singular values from Equation (5).

(6) $$S=\sum_{i=1}^{r}\sigma_{i}^{\prime }\mu_{i}v_{i}{}^{H}$$

In Equation (6), the left and right eigenvectors of the matrix are represented by $\mu_{i}$ and $v_{i}$, respectively, and $\sum_{i}^{}\sigma_{i}\mu_{i}v_{i}{}^{H}$ denotes the i subspace echo signal. By rewriting Equation (6) after vector subspace decomposition, we obtain

(7) $$S=\sum_{i\in T}^{}\sigma_{i}\mu_{i}v_{i}{}^{H}+\sum_{i\in N}^{}\sigma_{i}\mu_{i}v_{i}{}^{H}$$

In Equation (7), T represents the set of target signal subspace, while N represents the set of clutter signal subspace. In the SVD-based clutter suppression technique, once the target and clutter subspaces have been divided, the corresponding singular values can be used to identify their respective subspace signal vectors. By setting the singular values corresponding to the clutter dominant subspace to zero, the corresponding clutter signal of subspace N can be effectively removed, thereby achieving the goal of clutter suppression. However, in practical applications of SVD filtering, accurate division of the signal subspace and clutter subspace can be a challenge, leading to inadequate removal of the clutter subspace vector. To address the issue of inaccurate signal subspace division in SVD-based clutter suppression, an improved method is proposed in this study. The new method is based on adaptive SVD, which involves processing the specific subspace after decomposition instead of simply removing the maximum singular value to suppress the clutter. By optimally adjusting the size of the corresponding singular value in the subspace, the SNR can be improved, leading to effective suppression of strong clutter.

Rewriting Equation (7) as Equation (8), where $W_{1}$ and $W_{2}$ denote the set of clutter in the first and second signal subspaces, $T_{1}$ and $T_{2}$ denote the set of target signals in the first and second signal subspaces. Both the direct wave and the probe-coupled reflected wave energies are much stronger than the target signal energy, and during the SVD process, the subspace is divided based on the energy ratio [28]. Thus, the strong clutter signal mainly exists in the subspace corresponding to the first singular value, while the target signal mainly exists in the subspace corresponding to the second singular value. Therefore, the first subspace echo signal contains a higher proportion of the interference signal, denoted as $W_{1}>T_{1}$, while the second subspace echo signal contains a higher proportion of the target signal, denoted as $T_{2}>W_{2}$. This approach enables the reduction of the first singular value by a factor of $k_{1}^{}$ to suppress strong clutter and the amplification of the second singular value by a factor of $k_{2}^{}$ to enhance the SNR of the target signal.

(8) $$S=(\sigma_{1}/k_{1})\mu_{1}\nu_{1}{}^{H}+(\sigma_{2}/k_{2})\mu_{2}\nu_{2}{}^{H}+\sum_{i=3}^{r}\sigma_{i}\mu_{i}v_{i}{}^{H}$$

The selection of the optimization coefficients $k_{1}^{}$ and $k_{2}^{}$ for the singular values is determined based on the image std (standard deviation), which ranges from 1 to $Q_{1}^{}/Q_{2}$ (*Q*_1_ is the first singular value, *Q*_2_ is the second singular value). The std reflects the degree of dispersion of the grayscale values of the image pixels around the mean value. If the image standard deviation is larger, it indicates that the grayscale values of the image pixels are more widely dispersed around the mean value, implying a better image quality. This means that the degree of separation between defects and background in the image is greater, resulting in a better suppression effect on the interference signal and a more optimal selection of the singular value optimization coefficients $k_{1}^{}$ and $k_{2}^{}$. The optimized experimental data, namely, the experimental data after clutter suppression, are subjected to 2D imaging. The standard deviation of the image is a function of the optimization coefficients $k_{1}^{}$ and $k_{2}^{}$. When the standard deviation of the image is the maximum, the values of $k_{1}^{}$ and $k_{2}^{}$ are selected most appropriately.

The std of image is calculated using the following formula:

(9) $$std=\sqrt{\frac{1}{MN}\sum_{i=1}^{M}\sum_{j=1}^{N}{\left(F(i,j)-u\right)}^{2}}$$

**Figure 3:**
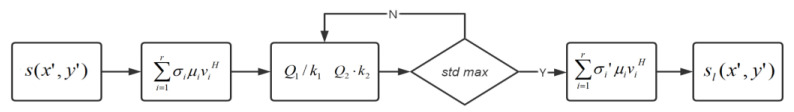
Clutter suppression method based on adaptive SVD.

In Equation (9), M and N represent the size of the image, F(i,j) represents the pixel value at row i and column j, and u represents the mean value, which is calculated by summing all pixel values and dividing by the total number of pixels in the image. When the maximum value of image std is obtained, the corresponding singular value optimization coefficients $k_{1}^{}$ and $k_{2}^{}$ are determined, and the new singular value matrix is reconstructed with the corresponding singular vectors to derive the signal $s_{l}(x^{\prime },y^{\prime })$ in the corresponding spatial coordinates after clutter suppression. Figure 3 illustrates the flowchart of the adaptive SVD method.

### 2.2. 2D Imaging Method Based on Spectral Reconstruction

To achieve highly accurate detection results, it is crucial to obtain a high-resolution, 2D image with clear target features. Currently, numerous scholars have devoted significant efforts to achieve high-quality 2D imaging by enhancing techniques, such as backprojection algorithms [29], frequency scaling algorithms [30], and holographic imaging algorithms [31]. The objective is to improve the signal-to-noise ratio and enhance image features. Among the various microwave imaging algorithms available, the spectral reconstruction algorithm stands out because it does not require any approximation conditions in the inverse reconstruction process [32,33]. By accurately focusing on the entire imaging area based on the scattering point model, the spectral reconstruction algorithm is designed to enable 2D imaging of defects inside GFRP.

The fundamental concept underlying the spectral reconstruction imaging algorithm is derived from Equation (3). Specifically, this process can be modeled as a plane wave passing through a linear, spatially-invariant system, which acts as a linear, dispersive spatial filter with a finite spatial bandwidth. In this filter, the amplitude of the wave remains constant within the spatial frequency domain passband, while a phase shift is introduced. Therefore, the fundamental principle underlying the spectral reconstruction imaging algorithm involves applying phase compensation to microwave signals in the spatial wave number domain [34,35]. This approach enables the algorithm to solve for the target image function g(x,y) at the location of the defect. Specifically, the filtered sampling data $s_{l}(x^{\prime },y^{\prime })$ can be reconstructed using the spectral reconstruction imaging algorithm to generate the image function g(x,y), thereby enabling the identification of the defect.

The algorithm involves several main steps. Firstly, the received reflected waves are converted from the spatial domain to the wave number domain using a 2D Fourier transform. Next, a matched filter is constructed to filter the spatial wave spectrum. Finally, the scattering characteristic distribution of the target is obtained by Fourier inversion, allowing for the reconstruction of the scattering intensity distribution at the target location.

Firstly, the sampled data after clutter suppression are transformed into the spatial wave number domain using the 2D Fourier transform, and the microwave spatial spectrum $S(k_{x},k_{y},z=R_{0})$ is derived:

(10) $$S(k_{x},k_{y},z=R_{0})=\int_{-L_{x}/2}^{L_{x}/2}\int_{-L_{y}/2}^{L_{y}/2}s_{l}(x^{\prime },y^{\prime })\exp[-j(k_{x}x+k_{y}y)]dx^{\prime }dy^{\prime }$$

In Equation (10), $L_{x}$ and $L_{y}$ correspond to the scanning range of the scanning plane, while $k_{x}$ and $k_{y}$ represent the spatial frequencies in the x and y directions, respectively.

Next, the spatial wave spectrum $S(k_{x},k_{y},z=R_{0})$ undergoes phase compensation, after which the spectrum $S(k_{x},k_{y},z=0)$ in the plane z=0 of the defect can be obtained:

(11) $$S(k_{x},k_{y};z=0)=S(k_{x},k_{y};z=R_{0})\times H(k_{x},k_{y})$$

In Equation (11), $H(k_{x},k_{y})$ is the transfer function of the linear dispersive spatial filter used for phase compensation. It compensates for the phase by equating the plane wave components propagating in different directions from the position $z=z_{0}$ to the position z=0. Its mathematical expression is as follows:

(12) $$H(k_{x},k_{y})=circ(\frac{k_{x}{}^{2}+k_{y}{}^{2}}{k^{2}})\exp(jk_{z}R_{0})$$

(13) $$k_{z}=\sqrt{k^{2}-k_{x}{}^{2}-k_{y}{}^{2}}$$

(14) $$circ(\frac{k_{x}{}^{2}+k_{y}{}^{2}}{k^{2}})=\left\{\begin{array}{ll} 1 & k_{x}{}^{2}+k_{y}{}^{2}<k^{2}\\ 0.5 & k_{x}{}^{2}+k_{y}{}^{2}=k^{2}\\ 0 & k_{x}{}^{2}+k_{y}{}^{2}>k^{2} \end{array}\right.$$

When calculating the transfer function, it is important to consider the distance between the defect in GFRP and the probe, denoted as $R_{0}+h$, as well as the distance between the defect and the surface of the GFRP workpiece, denoted as h. The transfer function is processed in the frequency domain, as shown in Equations (15)–(17), where the spatial wave number is k=4πf/c, f is the emitted signal frequency, c is the electromagnetic wave propagation velocity in the medium, and $k_{1}$ and $k_{2}$ are related to the air and GFRP dielectric constants, respectively.

(15) $$H_{1}(k_{x},k_{y})=circ(\frac{k_{x}{}^{2}+k_{y}{}^{2}}{k_{1}{}^{2}})\exp(j\sqrt{k_{1}{}^{2}-k_{x}{}^{2}-k_{y}{}^{2}}R_{0})$$

(16) $$H_{1}(k_{x},k_{y})=circ(\frac{k_{x}{}^{2}+k_{y}{}^{2}}{k_{2}{}^{2}})\exp(j\sqrt{k_{2}{}^{2}-k_{x}{}^{2}-k_{y}{}^{2}}h)$$

(17) $$H(k_{x},k_{y})=H_{1}(k_{x},k_{y})\cdot H_{2}(k_{x},k_{y})$$

Finally, the target scattering intensity at the corresponding spatial coordinate position, i.e., the target image function g(x,y), is obtained by performing the 2D Fourier inverse transform of the spectrum $S(k_{x},k_{y},z=0)$ at the target plane.

(18) $$g(x,y)=\frac{1}{4\pi^{2}}\int_{K_{x}}\int_{K_{y}}S(k_{x},k_{y},z=0)\exp(jk_{x}x+jk_{y}y)dk_{x}dk_{y}$$

The values of $K_{x}$, $K_{y}$ in Equation (18) can be determined by using the relationship between the independent variables of the Fourier transform pair:

(19) $$K_{x}=\pi /\Delta x^{\prime }\;K_{y}=\pi /\Delta y^{\prime }$$

**Figure 4:**
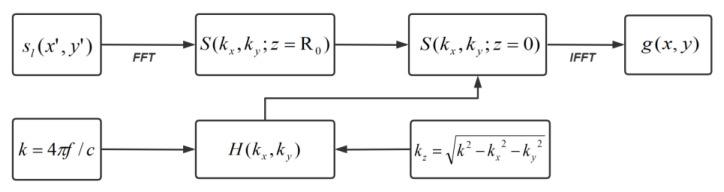
Spectral reconstruction algorithm.

The variables $\Delta x^{\prime }$ and $\Delta y^{\prime }$ in Equation (19) represent the step along the two coordinate axes. The flow of the spectral reconstruction algorithm is depicted in Figure 4.

### 2.3. Quantitative Characterization Methods

To enable the quantitative evaluation of defects, the image function g(x,y) must first undergo processing to extract the target defect region A from the background region B of the 2D image G. Next, statistical analysis is performed on image regionsg(x,y)[(x,y)∈A] containing only defects, allowing for the characterization of defect locations in terms of their region center coordinates, as well as the quantification of defect size in terms of region area.

To perform quantitative evaluation of defects, several steps are taken. First, the pixel values of the image function g(x,y) are normalized to obtain the function $g^{\prime }(x,y)$. Second, the optimal threshold T for image segmentation is obtained using the OTSU algorithm [31]. Next, $g^{\prime }(x,y)[(x,y)\in A]$ is binarized using this threshold to obtain the function g(x,y)[(x,y)∈A], which contains only the defective regions A. Finally, the center-of-mass coordinates $\left(x_{i},y_{i}\right)$ of each part of the defective regions are obtained using the Regionprops function, and the number of pixels in each defective region is determined based on the center-of-mass coordinates. This pixel count is then converted into the defective area $S_{i}$ to quantitatively characterize the defect location and area.

The OTSU algorithm, also known as the maximum between-class variance method, is based on the least squares principle and uses the grayscale histogram to achieve the best segmentation in a statistical sense. Its basic principle is to segment the grayscale value of the image into two parts using the best threshold so that the variance between the two parts is maximum, resulting in maximum separability. Let t be the segmentation threshold between the defect and the background. The number of defective pixel points as a proportion of the image is $w_{0}\left(A/G\right)$, with an average grayscale of $u_{0}$. Similarly, the number of background points as a proportion of the image is $w_{1}(B/G)$, with an average grayscale of $u_{1}$. The total average grayscale of the image is u, and v is the variance of the target defect and the background image:

(20) $$u=w_{0}\times u_{0}+w_{1}\times u_{1}$$

(21) $$v=w_{0}\times {\left(u_{0}-u\right)}^{2}+w_{1}\times {\left(u_{1}-u\right)}^{2}$$

Combining Equations (20) and (21):

(22) $$v=w_{0}\times w_{1}\times {\left(u_{0}-u_{1}\right)}^{2}$$

The primary objective of the algorithm is to identify the optimal threshold T among the threshold t variables, which maximizes the interclass variance v between region A and region B. To improve the efficiency of the algorithm, a two-step approach of integer traversal and fractional traversal is employed to find the optimal threshold value through stepwise approximation. Firstly, the pixel values $g^{\prime }(x,y)$ of the entire image region G are normalized to 255 levels of gray values. Then, the algorithm searches for the best integer threshold value $T_{N}$ within the range of 1 to 255 integers. Subsequently, the search continues in the range of $T_{N}-1$ to $T_{N}+1$. At this point, the difference between the defective region A and the background region B is maximum, and T is the best threshold value. When the variance v reaches its maximum, it can be inferred that the difference between the defective region and the background region is also at its maximum. Hence, the threshold value T obtained at this point is considered to be the optimal threshold. Finally, the threshold T is used to segment the image function $g^{\prime }(x,y)$ into $g^{\prime }(x,y)[(x,y)\in A]$, which are then binarized such that the pixel values in the defective region A are set to 1 and the pixel values in the background region B are set to 0. This process produces a 2D image function g(x,y)[(x,y)∈A] containing only the defects.

**Figure 5:**
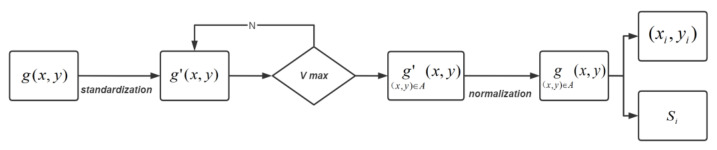
Quantitative characterization method.

To further achieve a quantitative characterization of the location and area of defects, the pixel statistics function of Regionprops is applied to g(x,y)[(x,y)∈A] in order to obtain the center-of-mass coordinates $\left(x_{i},y_{i}\right)$ and area $S_{i}$ of each region, thus enabling a quantitative characterization of the defects. Figure 5 illustrates the flow of the quantitative characterization method.

### 2.4. Complete Process and Step-by-Step Description

**Figure 6:**
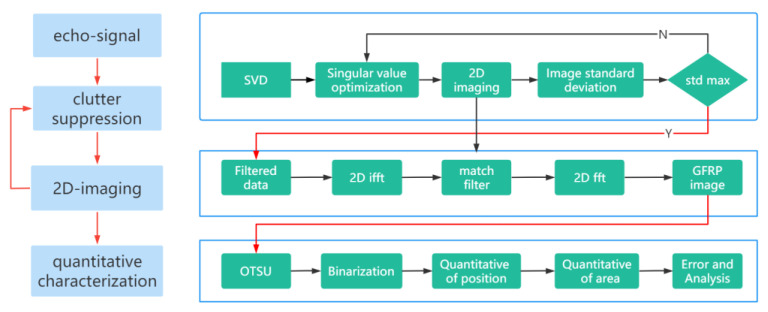
Complete process and step-by-step description.

The entire process can be divided into three steps: adaptive SVD clutter suppression processing, spectral reconstruction 2D imaging, and quantitative characterization based on the OTSU image segmentation algorithm. Figure 6 illustrates the overall flow chart of the process.

In the clutter suppression process, we first perform SVD decomposition on the experimental data to obtain the singular value matrix. Next, we optimize the first two singular values based on the image standard deviation. When the standard deviation reaches its maximum, we solve for the new singular value matrix using the corresponding optimization coefficients. Finally, we multiply and reorganize the singular value matrix with the left and right singular vectors to complete the adaptive SVD clutter suppression processing.

In 2D imaging, the data undergo a 2D Fourier inversion to transform the spatial domain into the wave number domain. A linear dispersive spatial filter is then constructed on the wave number domain to match the filtered signal. Finally, the matched filtered data are transformed back to the spatial domain by the 2D Fourier transform to complete the wave-spectrum reconstruction for 2D imaging.

In quantitative characterization, the image is segmented using the OTSU image segmentation algorithm to find the segmentation threshold of defects and background. The image is then binarized based on this threshold to obtain a two-dimensional image containing only defects. The Regionprops function is used to find the center-of-mass coordinates of each defect, and the defect area is calculated based on these coordinates to complete the quantitative characterization of defect location and area.

## 3. Experimental Verification and Analysis

### 3.1. System Construction and Data Acquisition

**Figure 7:**
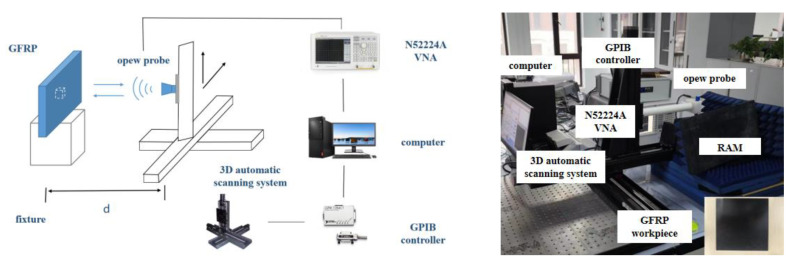
Microwave detection system.

As illustrated in Figure 7, we developed a 2D scanning microwave detection experimental system. This system comprises a vector network analyzer (VNA N5224A, from Agilent, Palo Alto, CA, USA), a Ka-band waveguide probe, a 3D automatic scanning stage, and automatic scanning control software. The VNA and waveguide probe are utilized to transmit and receive microwaves directionally in the microwave detection system. Additionally, the waveguide probe is moved by the 3D automatic scanning table to ensure that the microwave signal covers the surface of the specimen. The parameters of the automatic scanning control software are adjusted using a computer, and the experimental data are processed accordingly.

**Figure 8:**
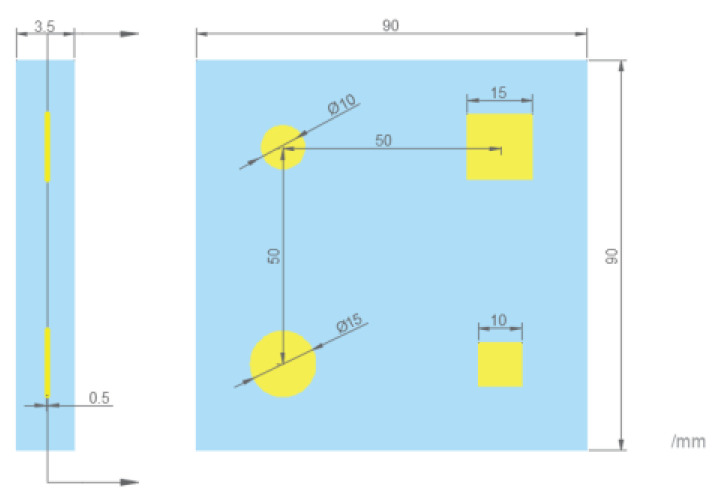
Design of GFRP specimen.

The experiment is conducted on a GFRP specimen with internal delamination defects. A 0.5 mm thick PTFE film is used to simulate the delamination defects, and Figure 8 presents information regarding the location and area of the defects.

To begin the sweeping experiment, it is necessary to determine the appropriate emission band. In order to achieve a balance between microwave penetration and resolution in the 2D scanning, we selected the Ka band (26.5–40 GHz) for our experiment. The distance between the waveguide probe and the surface of the specimen was set to 10 mm. If the lift-off is too small, the microwave signal cannot approximate a plane wave, which can affect the wave spectrum reconstruction into an image. Conversely, if the lift-off is too large, the waveguide probe receives less reflected signal. The automatic scanning range was determined based on the size of the workpiece and the location of the buried defects, and was set at 90 mm × 90 mm. In order to ensure efficient experimental scanning and accurate image reconstruction, it is crucial to determine a suitable scanning step. Typically, the scanning step should not be less than a quarter of the wavelength, which is calculated at the mid-frequency point of the Ka band, to avoid a decrease in scanning efficiency or a lack of data for image reconstruction. Lastly, the number of scan points was calculated based on the scan range and step distance, and set at 46 × 46. The experimental parameters are shown in Table 1.

### 3.2. Data Processing and Imaging

**Figure 9:**
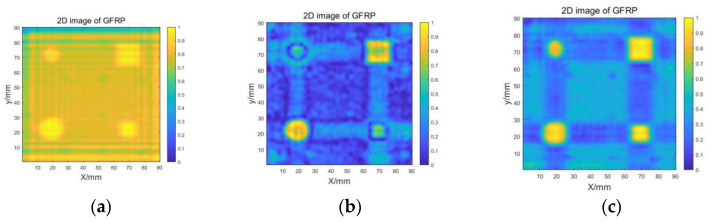
Comparison of imaging results: (**a**) without filtering; (**b**) traditional SVD method; (**c**) adaptive SVD method.

After collecting the experimental data, 2D imaging was performed using the spectral reconstruction algorithm without any clutter suppression processing. The resulting image, as shown in Figure 9a, exhibits streaks that interfere with the accurate quantitative characterization of the target defects. When the traditional SVD method is used for filtering, the imaging results are shown in Figure 9b. The defect features are not obvious and the contrast is poor. To address this issue, the echo signal was subjected to an adaptive SVD clutter suppression process to reduce the interference from clutter signals prior to 2D imaging. The developed algorithm is imaged under near-field conditions. However, it can theoretically also be applied to far-field imaging, but current research focuses on near-field imaging due to the significant impact of stand-off, resulting in relatively poor far-field results.

**Figure 10:**
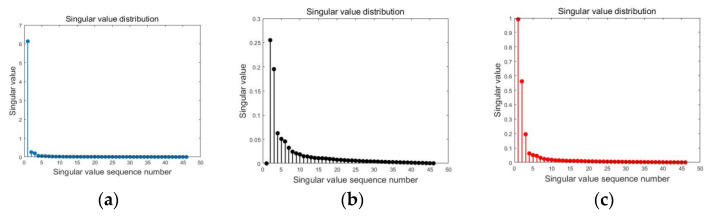
Singular value: (**a**) without filtering; (**b**) traditional SVD method; (**c**) adaptive SVD method.

In the adaptive SVD clutter suppression process, the first two singular values are optimized based on the method described in Section 2.1, and the corresponding std of the images with different optimization coefficients are computed. Figure 10c displays the results of the singular value optimization, with a maximum std of 0.167, corresponding to the first singular value optimization coefficient $k_{1}^{}$ of 7.2 and the second singular value optimization coefficient $k_{2}$ of 2.2. The optimized singular value matrix and singular vector are multiplied and recombined to obtain the clutter-suppressed echo signal, which is then used for 2D imaging using the spectral reconstruction algorithm. The imaging results are shown in Figure 9c. Compared to other imaging methods or clutter suppression techniques, our proposed combination of adaptive SVD and spectral reconstruction, along with techniques such as PCA [15] and SAR [16] imaging, offers superior imaging quality and contrast. These enhancements enable clearer visualization and differentiation of defects from the background, allowing for more precise measurements and evaluations.

### 3.3. Quantitative Characterization and Analysis of Results

**Figure 11:**
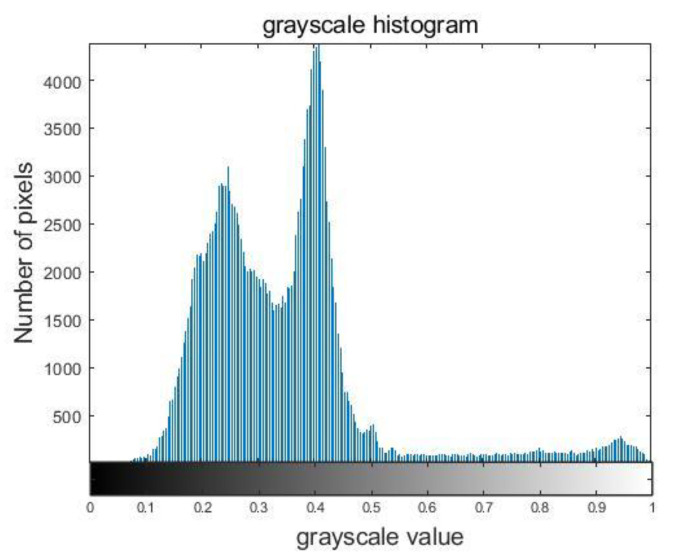
Grayscale histogram.

As illustrated in Figure 11, we conducted grayscale histogram statistics of the 2D image that was formed. Based on this analysis and the OTSU image segmentation algorithm, we determined the segmentation threshold T to be 0.561. To further evaluate the quality of the image, we introduced signal-to-background ratio (SBR) of image. This concept was derived from spectroscopy and measures the level of significance of the target signal. It provides an intuitive reflection of the ratio between the grayscale value of the target signal and that of the background [32]. Equation (23) is used to calculate this ratio, where $f_{s}(x,y)$ denotes the mean gray value of the signal region, and $f_{b}(x,y)$ denotes the mean gray value of the background region. In situations where the gray value of the signal is equal to that of the background, this indicates that the signal and background cannot be differentiated. As a result, a larger SBR indicates better image quality. Using the determined threshold value T, we can solve for SBR=4.41dB.

(23) $$SBR=10\left|\mathrm{lg}\frac{f_{s}(x,y)}{f_{b}(s,y)}\right|$$

**Figure 12:**
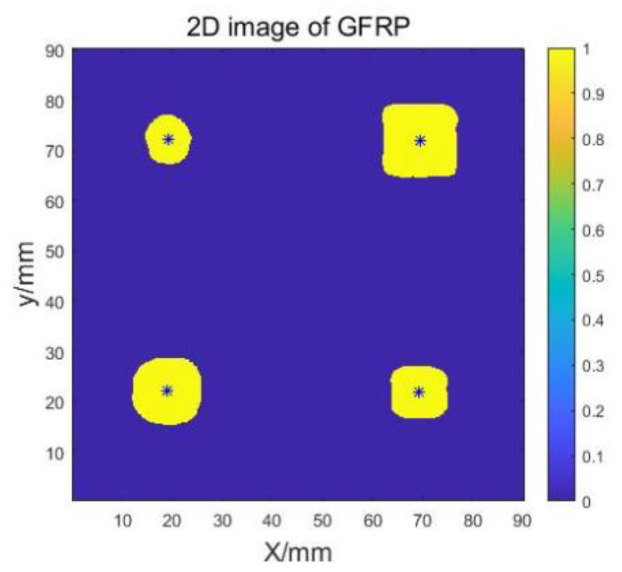
Delamination defects.

Based on the previously determined threshold value T, we performed further binarization of the GFRP image to eliminate the background information and obtain a 2D image that displays only the defects. The resulting image of delamination defects is presented in Figure 12, and it can be observed that the shapes of the defects depicted in the image are in good agreement with the anticipated design defect shapes. 

In order to characterize the number and location of defects, we conducted pixel statistics using the Regionprops function to extract the center-of-mass coordinates $\left(x_{i},y_{i}\right)$ for each defect present in the image. The resulting center-of-mass coordinates were then utilized to determine the relative location information of the defects, and the obtained results are tabulated in Table 2.

**Figure 13:**
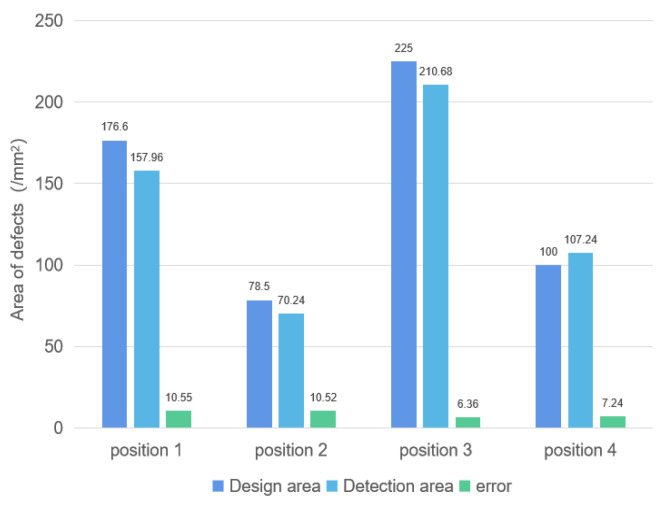
Area detection results.

To further characterize the defect area information, we determined the number of pixels for each individual defect part based on its center-of-mass coordinates. Given that the side length of each pixel block is 2 mm (scanning step), we were able to convert the number of pixels into an area measurement $S_{i}$. We compared the characterized area with the design area to evaluate the accuracy of our defect area characterization method. The resulting evaluation errors are presented in Figure 13.

From the characterization results, it is evident that the adaptive SVD clutter suppression process has greatly improved the accuracy of defect detection. The position detection error is within 0.5 mm, and the area detection error is within 11%, indicating a high level of accuracy. 

However, some errors were observed during the characterization process. These errors can be attributed to two main reasons. Firstly, there were processing errors in the specimen defect itself. During the preparation of the specimen, inaccuracies in the cutting size of the polyethylene film caused the actual area of the buried defect to deviate from the designed area. This led to errors in the characterization of the defect area. Secondly, experimental errors were introduced due to variations in the lifting-off of the waveguide probe at different locations on the GFRP specimen. These variations, combined with the inability to guarantee absolute perpendicularity between the specimen and the probe, resulted in inaccurate experimental sampling data.

## 4. Conclusions

In this paper, we proposed a nondestructive quantitative detection method for internal defects in GFRP. Firstly, the proposed method exhibited excellent imaging performance and effectively highlighted the features of defects. By comparing the 2D images before and after applying the improved clutter suppression method, the effectiveness of the proposed adaptive SVD clutter suppression method and spectral reconstruction algorithm was demonstrated. In addition, the proposed adaptive SVD clutter suppression method can be applied to detect other defects in GFRP using microwave detection. Secondly, the proposed method demonstrated high accuracy in quantitative evaluation. The quantitative detection results demonstrate that the quantitative characterization method is capable of defect detection without leakage, position detection with an error within 0.5 mm, and area detection with an error within 11%. 

In summary, the proposed method enables high-quality visual imaging and precise quantitative characterization. Future endeavors will focus on visualizing and quantitatively detecting defects with smaller spatial dimensions.

## Figures and Tables

**Table 1:** Experimental parameters.

Microwave Frequency Band	Stand-Off Distance	Scanning Range	Scanning Step	Scanning Points	Frequency Point of 2D Imaging
Ka (26.5–40 GHz)	10 mm	90 × 90 mm	2 mm	46 × 46	39.5 GHz

**Table 2:** Position detection results.

	Position 1	Position 2	Position 3	Position 4
Theoretical (mm)	(0, 0)	(0, 50)	(50, 50)	(50, 0)
Detection (mm)	(0, 0)	(0.17, 49.93)	(50.41, 49.80)	(50.27, −0.28)
Error (mm)	(0, 0)	(0.17, 0.07)	(0.41, 0.20)	(0.27, 0.28)

## Data Availability

The data presented in this study are available on request from the corresponding author after obtaining permission of an authorized person.
